# Clinical applications of avian eggshell-derived hydroxyapatite

**DOI:** 10.17305/bjbms.2020.4888

**Published:** 2020-11

**Authors:** Horia Opris, Simion Bran, Cristian Dinu, Mihaela Baciut, Daiana Antoaneta Prodan, Alexandru Mester, Grigore Baciut

**Affiliations:** 1 Department of Maxillofacial Surgery and Implantology, “Iuliu Hatieganu” University of Medicine and Pharmacy, Cluj-Napoca, Romania; 2 Department of Oral Health, “Iuliu Hatieganu” University of Medicine and Pharmacy, Cluj-Napoca, Romania

**Keywords:** Bone tissue regeneration, eggshell, guided bone regeneration, hydroxyapatite, socket preservation

## Abstract

The search for bone reconstruction materials and methods is an ongoing challenge. The aim of this review is to systemically search the available literature concerning the clinical performance of eggshell as a substitute material in guided bone regeneration in oral surgery. Five databases (PubMed, Cochrane, Web of Science, Scopus, and Embase) were searched up to February 2020. Clinical trials that used eggshell as a bone substitute material were included in the review. Animal and *in vivo* studies were excluded from the review. ROBINS-I was used to evaluate the risk of bias. A total of 840 studies were retrieved, out of which 55 full-text articles were screened. Five studies were finally included: one study showed critical and four serious risk of bias. A total of 74 patients and 88 intervention sites were included in the five studies. Clinical and radiological evaluation showed complete healing during the follow-ups. Statistically significant radiological and clinical evidence of new bone formation was achieved for socket preservation, grafting after third molar extraction, and cystic/apicectomy grafting. One patient with complications was reported. Histological analysis and micro computed tomography confirmed that it promotes bone regeneration. A comparison with synthetic hydroxyapatite showed similar healing characteristics. Within the limitations of the included studies, the eggshell can be safely and efficiently used in guided bone regeneration procedures, but more research is needed to completely evaluate the full potential of this material.

## INTRODUCTION

Bone regeneration is a major challenge in reconstructive surgery [1]. The gold standard for alveolar reconstruction is the autologous bone, enhancing osteogenesis, osteoinduction, and osteoconduction [2]. The problem lies in its scarcity, depending on the donor site, and association with several risks like nerve damage, infection, bleeding, scarring, and loss of function [3]. New materials that strive to overcome these shortcomings have emerged. Their success is limited due to the scarcity of viable osteoblasts [4]. The oral cavity is a unique environment due to the presence of bacteria and saliva, and also due to the mastication that occurs there. It is very important to take all these into consideration when searching for a suitable biomaterial to be used [5].

Unlike autografts, allografts consist of tissue transferred from another individual of the same species [6]. One of their most common drawbacks is issues with tissue integration and vascularization [7]. The use of allografts is particularly advantageous when donors are rare, and their supply is theoretically unlimited. Another concern is the probability of disease transmission through the material. However, the risk is minimal when donor selection protocols are well established [8]. Freeze-dried bone is the most frequently produced byproduct. It is used in the following two ways: demineralized allograft (DFDBA) or mineralized allograft (FDBA) [9].

Tissues obtained from other species are called xenografts. Hydroxyapatite (HA) and bovine grafts are the most representative xenograft materials [10]. The main advantage of these materials is that they are osteoconductive grafts which serve as a matrix for bone regeneration. They have low resorption rates. The disadvantages of xenografts are their high risk of an immune reaction, friability, and the fact that they easily migrate [9].

Alloplastic materials are synthetic graft materials [11]. Tricalcium phosphate (TCP), calcium carbonate and sulfate, bioactive glass, and HA are among the most used alloplastic graft materials. Just like xenografts, they provide a matrix for bone regeneration through osteoconduction [9].

Guided bone regeneration (GBR) is a concept that separates two distinct spaces where osteogenic cells are exclusively allowed to populate a bone defect, and the non-osteogenic cells are prohibited from invading the desired area [12]. Membranes are used extensively for GBR. There are resorbable and non-resorbable membranes [13]. An ideal membrane has to be biocompatible and semi-permeable, with good mechanical and physical proprieties [14]. Titanium and polytetrafluoroethylene (PTFE) are non-resorbable materials used in such procedures. Their main advantage is the ability to maintain their volume for new bone formation [15]. Their biggest setback is that they need surgical reentry for removal, and the most frequent complications include infections and membrane exposure. Resorbable membranes do not require reentry, but they do not have the mechanical proprieties of the non-resorbable ones. Similar results of the achieved bone quality are reported for these membranes [16].

An eggshell is composed of three layers which are: the cuticle, spongia, and lamella [17]. These contain protein fibers and calcium carbonate. The weight of the eggshell accounts for less than a third of that of the egg, and it is composed mainly of calcium compounds (over 90%) and small traces of organic matter [18].

The eggshell is a great source of calcium for dietary supplementation [19]. It has been shown to reduce bone loss in postmenopausal women and patients with osteoporosis [20]. It is also used as a matrix for bone formation in animal models [21]. The eggshell membrane is also extensively used as a dietary supplement, being beneficial to joints and regeneration of connective tissue. The eggshell membrane was also used in various experimental studies to assess its bone formation capabilities [22,23]. Recent developments have tried to associate nanohydroxyapatite into different compounds to enhance its proprieties even more [24].

Hence, the purpose of this paper is to evaluate the clinical performance of the eggshell in enhancing bone regeneration in alveolar bone defects.

## MATERIALS AND METHODS

### Protocol development and reporting format

The review method was elaborated per the PRISMA guidelines [25]. Following the PICO criteria (Population, Intervention, Comparison, Outcome), the articles included had to meet the following criteria:

- Population: patients ≥18 years old, without systemic diseases, in need of bone regeneration interventions, and non-smokers.
- Intervention: any given intervention for oral bone regeneration.
- Comparison: any given intervention for oral bone regeneration in controlled studies.
- Outcome measures:
  ◾ primary outcome – changes in the clinical height, width, density, and healing time of the alveolar ridge by linear measurements between baseline and follow-up.
  ◾ secondary outcome – surgical complications, changes in the marginal bone level, patient-reported outcome measures, and adverse effects.

The research aimed to respond to the following focused questions:

- In healthy patients, is the use of eggshell efficient to enhance the GBR process?
- Does the eggshell ensure better/faster bone regeneration?
- Is the eggshell a sustainable bone regeneration substitute?

### Eligibility criteria

Inclusion and exclusion criteria were defined. Regarding the study design, randomized clinical trials (RCTs), non-RCTs, and prospective control clinical trials were considered eligible.

Studies with the following criteria were included: participants ≥18 years old, available clinical and radiological examination, cases of unrestorable teeth with periapical lesions/cysts/impacted third molars, GBR surgical interventions using the eggshell, follow-up until complete gingival and bone healing, non-smoker participants, participants with no systemic diseases.

The following categories of studies were excluded: studies with participants with poor oral hygiene; pregnant participants. Abstracts, letters to the editor, narrative reviews, case reports, case series, technical notes, position papers, and articles with unclear or insufficient information for data quantification were also excluded from the study.

### Research databases and screening

A search was conducted on five databases (PubMed, Web of Science, Cochrane, Scopus, and Embase) to find all human papers published in English up to February 2020, using the following keywords: “eggshell” with the following terms “bone regeneration,” “GBR,” “osseointegration,” “tissue regeneration,” “bone graft,” “bone healing,” “bone biology,” “bone substitute,” “bone repair,” “bone health,” “bone metabolism.” Articles were screened by two independent reviewers (H.O. and A.M.). The assessment for eligibility was firstly done by reading titles and abstracts to remove duplicates. Secondly, articles meeting the inclusion criteria were assessed in full. Any difference in opinion between the assessors was resolved through deliberation, and in case a conclusion was not established, a third reviewer (D.A.P.) was solicited.

### Data collection

Characteristics of the included papers were determined by two reviewers (H.O. and A.M.). The following features were noted: author, year of publication, country of origin, study design, study period, main objectives, participants, inclusion criteria, exclusion criteria, type of material used, intervention site, clinical and radiological assessment, biopsy (histology), follow-up, complications, and outcome.

### Risk of bias assessment

ROBINS-I [26] was utilized for the quantification of the risk of bias in seven domains: confounding, included participants, types of interventions, deviations from the intended interventions, missing data, and measurements of results bias in the selection of the reported outcomes. The judgment of the bias was evaluated as follows: low (low risk for all fields), moderate (low/moderate for all fields), serious (serious risk in at least one field, but not critical in any field), critical (critical risk in at least one field), and no information (no clear evidence that the study is at risk and there is a lack of data in one or more key fields) [26]. Two reviewers (H.O. and A.M.) separately evaluated the risk of bias for these studies, and if any disagreement occurred, a third reviewer (D.A.P.) intervened.

### Ethical statement

In this study, humans were not involved. We did not need ethical approval.

## RESULTS

### Study selection

**FIGURE 1:**
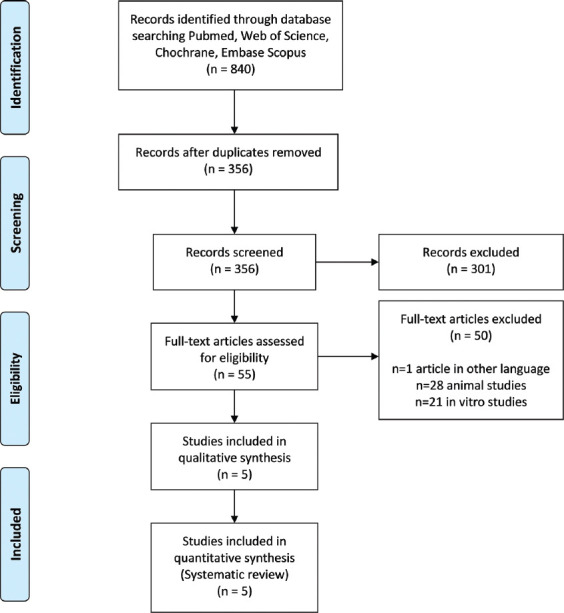
PRISMA flow diagram.

The electronic search yielded 840 articles that were reduced to 356 after the removal of duplicates (Figure 1). No further articles were identified by manual search. Screening of titles and abstracts led to the exclusion of 301 records. The whole texts of the remaining 55 papers were retrieved. The reviewers further excluded 50 studies. The full texts of the five remaining papers were analyzed systematically and quality wise (Table 1).

**TABLE 1:**
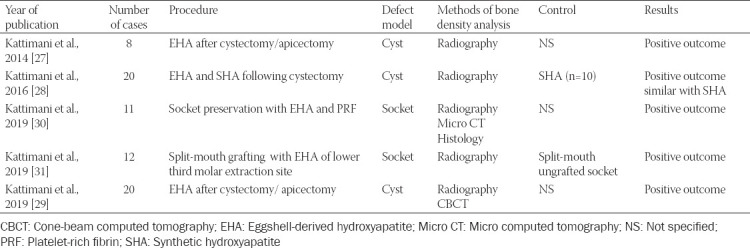
Characteristics of the clinical trials included

### Study characteristics

All included articles were non-RCTs (n = 4) [27-30], except for one RCT [31]. One study employed a split-mouth design [31], one study used two parallel groups (n = 10) [28], with the remaining three studies being pilot studies [27,29,30]. The number of patients per study ranged from 8 to 21, with a total of 74 included in this analysis.

The RCT compared the efficacy of the eggshell to that of the control site [31], one non-RCT compared the efficacy of the eggshell to that of HA after cystectomy [28], one used the eggshell in socket preservation [30], and the remaining two non-RCTs used the eggshell in regeneration after cystectomy/apicectomy [27,29].

All studies presented follow-up of gingival and bone healing at 3 months [27], 4 months [29,30], or 6 months [28,31]. In all the included studies, patients were followed-up through clinical evaluation and imaging. For one study covering 24 weeks [31], a biopsy was taken using trephine bur, then micro computed tomography and histological assessment were provided subsequently. Only one complication (wound dehiscence) was reported [29], and two other patients were excluded from the study due to the lack of follow-up [31].

### Primary outcomes

Overall, five articles were included with 74 patients and 88 intervention sites. The evaluation of the primary outcome intended to find out if there were any changes in the dimensions, as well as the density and healing time of the alveolar ridge, by comparing measurements between the baseline and follow-up procedure after using the eggshell as bone graft material.

### Radiological surgical site outline

Only four of the studies evaluated the limits and the surgical site outline (the contour of the augmented area), referred to as the lamina dura in some of them. For third molar extraction sites, no significant difference (*p* < 0.05) in the lamina dura was seen as opposed to the control group at 1-month and 6-months follow-up in a split-mouth model [31]. The comparison of the radiological assessment of the operation site outline in apicectomy and cystic defects (time intervals: 1^st^, 4^th^, 8^th^, 12^th^, and 24^th^ week) showed a significant decrease in healing time (*p* < 0.05) while the union of the graft and surgical site was complete by 24 weeks [29].

When comparing the surgical site of the synthetic HA (SHA) and eggshell-derived HA (EHA) groups, there was no significant difference in the lamina dura between the two at 1, 2, 3, and 6 months [28]. The comparison of the surgical site outline for cystic and apicectomy defects at the 1^st^, 4^th^, 8^th^, and 12^th^ week showed a statistically significant decrease in the lamina dura with time (*p* < 0.05) [27].

### Radiological bone formation

In socket preservation with the eggshell and platelet-rich fibrin (PRF), 73.91% of sockets healed in a trabecular pattern, while the remaining 26.09% were found to present a ground-glass appearance [30]. Histological evaluation showed adverse effects due to the bone regeneration procedure [30].

Bone formation at 1, 3, and 6 months was compared between the control versus graft in third molar extraction site and showed no significant difference (*p* > 0.05) [31]. The eggshell group showed over 80% trabecular bone at the final evaluation compared to the empty socket group, where only half of the bone healed in a trabecular pattern [31].

Comparison of the radiological evaluation of bone healing at distinct time intervals (1^st^ vs. 4^th^, 4^th^ vs. 8^th^, 8^th^ vs. 12^th^, and 12^th^ vs. 24^th^) using the eggshell in cystic and apicectomy defects over the time intervals showed significant differences between the compared intervals (*p* < 0.05) [29]. Complete radiological bone healing was visible with compact bone (90%) and 10% trabecular bone at 8 weeks (*p* < 0.05) [29].

Comparing HA and eggshell radiological evaluation of the bone formation and bone healing patterns showed no significant difference at 1, 2, 3, and 6 months (*p* > 0.05) [28]. The HA and eggshell presented similar radiological bone healing patterns [28]. When comparing radiological bone formation using the eggshell at different time intervals (1^st^, 4^th^, 8^th^, and 12^th^ week) in cystic cavities, significant amounts of new bone can be found with time (*p* < 0.05) [27].

### Mean bone radiological density

Comparison of the density scores for the socket preservation sites with nanohydroxyapatite (eggshell) and PRF in the 1^st^, 12^th^, and 24^th^ week showed a significant increase in bone mineralization (*p* < 0.05) [30]. The radiological analysis of bone density at baseline, 1^st^, 3^rd^, and 6^th^ month in two wisdom molar extraction groups concluded that there was no significant difference in the bone density (*p* > 0.05), except for the total density in the control versus graft at 1^st^ month (*p* = 0.0492) [31].

No significant difference (*p* > 0.05) was found when comparing the mean density of bone of HA versus eggshell-derived HA at 1^st^–6^th^ month, with the exception of the comparison between the 1^st^ month and the 6^th^ month [28]. Another evaluation of the mean bone density at 1^st^, 4^th^, 8^th^, and 12^th^ week showed a significant increase in the bone density with time (*p* < 0.05) [27].

### Secondary outcomes

The following were assessed as secondary outcomes: surgical complications, changes in the marginal bone level, patient-reported outcome measures, and adverse effects. Only one patient with a complication (wound dehiscence) was reported [29]. The marginal bone level was reportedly maintained in all studies for the specific follow-up time frames. No published study reported adverse effects such as disease transmissions or allergic reactions.

### Other features examined

One study compared the initial and final alveolar width after socket preservation with the eggshell-derived HA and PRF. It showed that using the eggshell can maintain a significantly better bone width with time, i.e., 14.04 mm ± 0.86 mm versus 13.48 mm ± 0.80 mm (mean difference 0.57 mm ± 0.43 mm) (*p* = 0.0001) [30]. Another study managed to compare probing depth in a split-mouth model after extraction of the third molar and grafting, with promising results (initial probing depth 2.19 mm ± 0.63 mm in the control group; 2.00 mm ± 0.68 mm graft group; probing depth at the end of follow-up: 1.14 mm ± 0.33 mm control; and 1.0 ± 0.19 mm graft group). The results showed good periodontal health, within normal limits [31]. Only one patient was reported with a surgical complication, a woman with wound dehiscence, who was excluded from the study [29].

## DISCUSSION

The present systematic review aimed to assess the clinical benefits of using an eggshell-based material in alveolar bone defect reconstruction interventions. In recent years, a great amount of research and development has focused on finding new materials for tissue regeneration, more exactly GBR. Human research has been conducted using the eggshell, with the first published study in 1998 in France [32], and continued with the group from India several years later (2014, 2016, and 2019) [27-31]. To increase the relevance of this review, we comprehensively conducted multiple database searches and included all available human studies with the eggshell as bone regenerative material used in oral surgery.

The main objective of this review was to see if the eggshell is efficient in enhancing the GBR process in healthy patients. In several studies, the eggshell can also be found as a nanohydroxyapatite composite due to its smaller-sized particles. Based on the results of this review, it was concluded that the lamina dura or the so-called surgical site outline fades away with time when using the eggshell as a bone substitute [27,29], similar to SHA [28]. At the end of the follow-up in a split-mouth model after the extraction of the third molar it was similar to the control site [31]. This process is indicative of bone healing, bone remodeling, and bone regeneration. It seems that it is neither faster nor slower than in bone healing [31].

The eggshell produces mostly trabecular bone with rates ranging from 73.91% [30] to 90% [29]. One study showed a significant improvement in the formation of new trabecular bone compared to a control group [31]. When compared with SHA, it shows no difference in bone pattern [28]. Bone density increased with time, though the difference is not significant when we compared it to a control group [31] or to HA [28].

One of the studies proved that the eggshell could maintain a higher alveolar width with time in a socket preservation model [30]. The probing depth showed good periodontal health with no periodontal pockets formed [31]. Few complications were reported; only one patient was excluded due to a surgical complication (wound dehiscence) [29].

One of the limits of this review is that it only included studies written in English. The first pilot study with the eggshell bone regenerative material was carried out by Baliga et al. in 1996 in France [32]. This study was excluded from this review. Another limitation is the fact that all the studies included have been done in the same center by the same team in different years [27-31]. When the risk of bias was evaluated, we discovered that there was a serious bias in all studies because patients were not recruited consecutively. They also needed to be present in all follow-ups and were excluded if any complications occurred. No data about the evolution of the excluded patients was published in any of the studies, with the exception of 2 patients, one excluded due to the lack of follow-up [31] and the other due to surgical complications [29]. One of the studies stated that patients with complications were excluded but did not state the number of patients and reason(s) for their exclusion [28].

The first pilot study by Kattimani et al. in 2014 [27] was considered to have critical bias concerning the selection of the patients, due to the fact that no inclusion or exclusion criteria were clearly defined. The study stated that there were two undefined groups of patients. This study also stated that all patients with complications due to loss of the graft or infection were excluded. No data about these patients were available.

**FIGURE 2:**
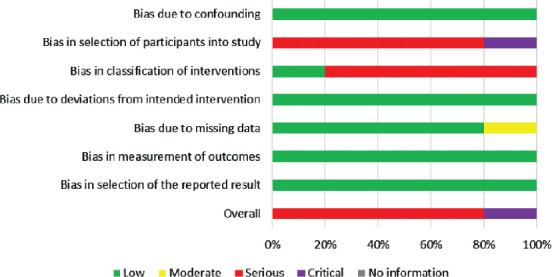
Overview of the risk of bias using the ROBINS-I tool.

After evaluating the risk of bias [26], we found that four out of the five studies presented serious, and one presented a critical risk of bias (Figure 2). To be able to review the current literature and the current state of knowledge, we had to include every possible study in this very narrow field of research, even if there were serious and critical concerns regarding their bias.

Recent studies pointed out that the ideal treatment for cystic bone lesions still remains enucleation and primary wound closure without any bone grafts [33]. This questions the results and the quality of the eggshell-derived biomaterial as a bone substitute in the included studies [27-29] due to the fact that the model uses cystic/apicectomy bone defects. No clear conclusion can be drawn using this kind of study protocol, as the lesion tends to heal spontaneously after enucleation with primary wound closure.

Due to the design of the socket preservation model using eggshell-derived biomaterial alongside PRF [30] we cannot conclude one of the materials or the other is efficient. PRF is widely used for guided tissue regeneration [34] and even bone regeneration [35] in some instances. Thus, it is difficult to conclude that the eggshell alone accounted for the results in that study.

Only one of the studies [31] used a design that excluded inter-individual variability with its split-mouth model for grafting the socket after the third molar extraction. This was the only RCT eligible for this review.

The protocols with which the patients were assessed were not uniform in the corresponding studies, although all the included studies were performed by the same team. Only one of the studies [30] mentioned inserting dental implants at 24 weeks, but there were no data available on the number, survival rate, failure rate, and complications. None of the studies mentioned patient-reported outcomes.

Prospective randomized clinical studies are required to assess without a doubt the viability of the eggshell as a regenerative material. A clear and well-defined surgical and follow-up protocols need to be developed to address the current limitations. Comparative and blind assessment of the interventions can be a good addition to the future developments.

One of the studies cited dental implant placement after the eggshell-based bone reconstruction but did not provide any additional information. Another future prospect may be the evaluation of the mastication forces in the bone surrounding dental implants, and their survival rates and viability compared to the today’s industry standard.

It is very important for the current practice to comprehend the failures, the reasons behind, and the mechanisms that produced them. The lack of solid data on complications and failures is another big drawback of the review that can only be counteracted through well-developed protocols and the inclusion of all available data. Due to the differences in the available data, this review was not able to statistically evaluate the studies included in the meta-analysis. The eggshell should be used in the future research with different types of membranes in GBR procedures. Another prospect for the future can be the use of the eggshell with complex dental implant surgery protocols for ridge augmentation and simultaneous implant placement, sinus lift, lateral or vertical augmentation, or even in combination with other materials (autologous bone and xenograft).

Upcoming studies may use other processing methods of the eggshell, like surface-modified carbonated apatite [36,37], eggshell microparticle (ESP) reinforced gelatin-based hydrogels [38], or eggshells from other sources like ostriches [39]. Another possibility would be to combine eggshells and bioactive materials such as recombinant human bone morphogenetic protein-2 (rhBMP-2), as it was done in other recent studies [40,41].

Future development includes a lot of active research on the potential therapeutic effect of the eggshell. This research includes eggshell processing as cement [42] and as a drug delivery system [43]. Studies using other core components of the eggshell are promising, including osteopontin [44] and lysozyme with its antiseptic proprieties, and even as membranes [45].

## CONCLUSION

Within the limits of this review, the eggshell as a bone regeneration material demonstrated, in different circumstances (socket preservation, cystectomy/apicectomy bone defect, and third molar extraction), that it can be a viable filler material in bone regeneration procedures in oral surgery. It is also inexpensive, readily available, and easy to produce. No diseases and no immunological responses were seen in the included patient groups. It also does not disturb the healing process of the soft and hard tissues. It can be efficiently used standalone or in combination with PRF in alveolar preservation procedures.
